# Intraocular Lymphoma or Infection? Subretinal Aspirate Confirms the Diagnosis

**DOI:** 10.1155/2018/6437603

**Published:** 2018-07-02

**Authors:** A. Saad, S. O. Salceanu, K. Oswal, A. Aboushrkh, D. Hamada

**Affiliations:** ^1^James Cook University Hospital (JCUH), Middlesbrough, UK; ^2^Zagazig University, Zagazig, Egypt

## Abstract

**Purpose:**

To demonstrate the importance of subretinal biopsy to reach a diagnosis when vitreous biopsy is negative or inconclusive.

**Methods:**

A 54-year-old Caucasian gentleman presented with bilateral anterior uveitis at JCUH. He initially responded to topical steroids and dilating agents. Subsequently he developed bilateral panuveitis and cataract with poor response to treatment. Detailed workup had been done to rule out infectious etiology. A suspicion of lymphoma was considered and vitreous biopsy sample was taken from one eye, which was inconclusive. Then, to help with definitive diagnosis vitreous sample, subretinal aspirate and retinal biopsy were taken.

**Results:**

Subretinal aspirate revealed* Aspergillus niger*. Treatment was initiated accordingly.

**Conclusions:**

Subretinal aspirate and retinal biopsy can help with diagnosis of unusual clinical panuveitis like presentation.

## 1. Introduction

Anterior uveitis, posterior uveitis, and panuveitis are most commonly diagnosed, investigated, and managed medically. Surgical interventions such as vitreous biopsy are occasionally necessary to aid in diagnosis and management of panuveitis. We present a rare case of panuveitis, where vitreous biopsy did not yield conclusive results, but subsequent subretinal aspirate grew a microbe!

## 2. Case History

**Figure 1:**
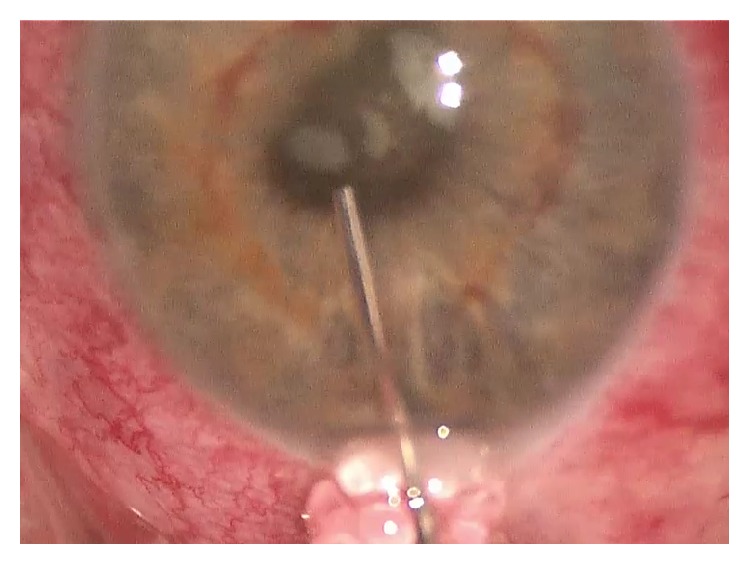
Total posterior synechia with seclusion pupillae and fundus obscuring cataract.

**Figure 2:**
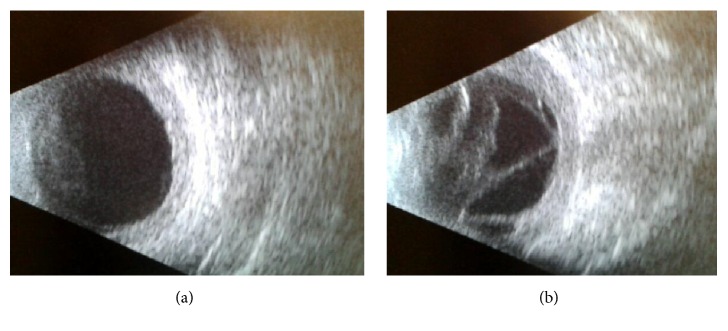
((a) and (b)) B scan US of right eye and left eye.

**Figure 3:**
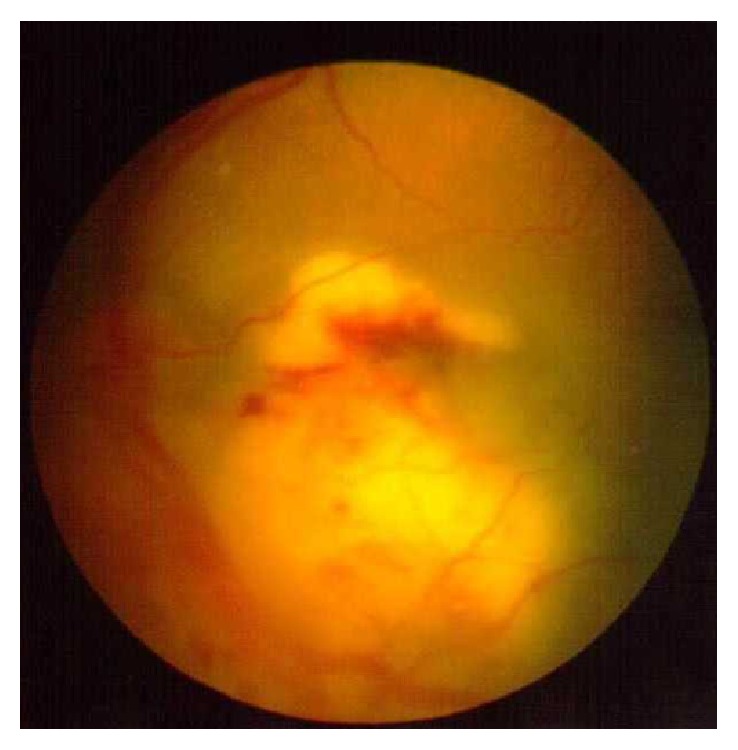
View through vitrectomy of the first eye.

**Figure 4:**
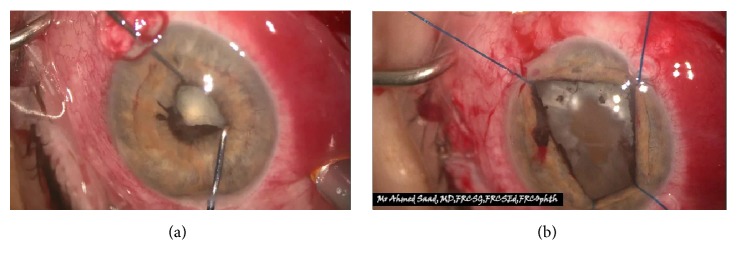
Synechiolysis and iris stretching prior to phacoemulsification with iris hooks & vitrectomy of the second eye.

**Figure 5:**
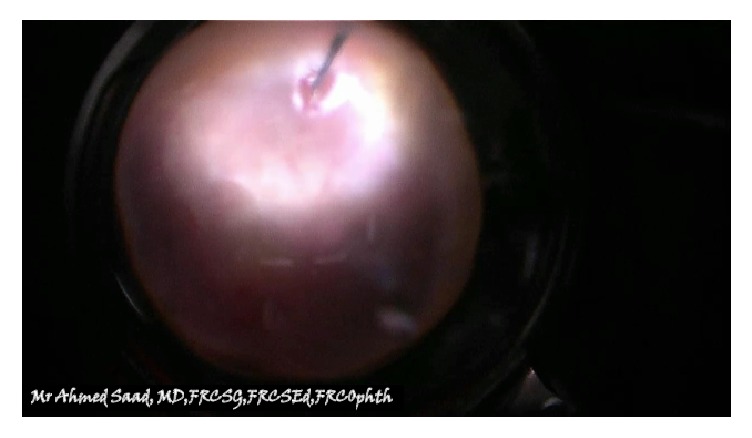
Subretinal biopsy through vitrectomy.

**Figure 6:**
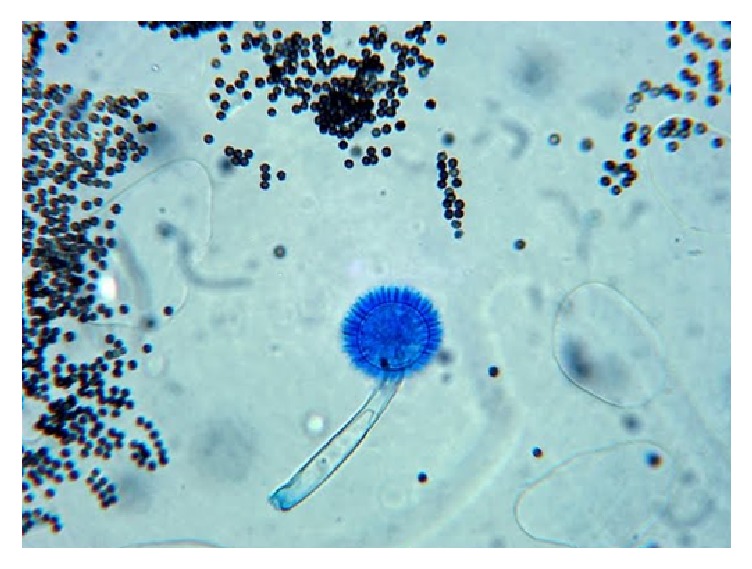
*Aspergillus niger* growth.

- 54-year-old immunocompetent Caucasian male presented with bilateral anterior uveitis with initial partial response to topical steroids.
- Few months later, this progressed to panuveitis with fundus obscuring cataract, total posterior synechia, seclusion pupillae (Figure 1), and poor response to treatment. His visual acuity quickly deteriorated to PL in both eyes.
- A detailed workup was done to rule out infectious etiology including TB, Syphilis, toxoplasma, and Herpes. All investigations came back negative.
- Further systemic workup was done to exclude autoimmune aetiology. All autoimmune screens (ANA, ANCA, and rheumatoid factor) came back negative.
- B scan showed diffuse choroidal thickening with mild exudative detachment and a diagnosis of lymphoma was considered (Figures 2(a) and 2(b), right eye and left eye, resp.).
- Phacovitrectomy and vitreous biopsy from one eye (Figure 3) were inconclusive as they showed atypical lymphocytes with no microbiology growth.
- Phacovitrectomy (Figures 4(a) and 4(b)), vitreous biopsy, and subretinal biopsy (Figure 5) were done for the second eye. The sample was sent in CytoLyt medium for cytology, which excluded lymphoma and pointed to numerous cocci-like organisms.
- Culture of another subretinal sample yielded* Aspergillus niger *finally (Figure 6). The patient was treated with intravitreal amphotericin B (dose: 5 micrograms/0.1 ml). Uveitis bilaterally improved with vision improvement from PL to CF.
- The patient was immunocompetent and investigations for HIV were negative; however, he admitted IV drug abuse, which could be the only risk factor found. Pulmonary aspergillosis was excluded by the chest physicians. Also, systemic workup by the medical team did not reveal any other systemic fungal infection.
- Subsequent follow-up showed improvement of vision to 2/60 with subretinal scarring. The site of subretinal retinal biopsy healed with a chorioretinal scar. Retina remained attached with no subsequent procedures performed.

## 3. Discussion

*Aspergillus niger* endogenous endophthalmitis is an extremely rare finding in immunocompetent patients with no history of lung aspergillosis. Our case was a fit and healthy immunocompetent patient with rapidly progressing bilateral panuveitis and fundus obscuring cataracts. Boldrey EE reported bilateral* Aspergillus* eye infection from metastatic heart valve infection [1]. This case was treated with vitrectomy and amphotericin B injection. Jager et al. [2] reported* Aspergillus niger* as a cause of endophthalmitis and scleritis.

There are other reports about the same organism as a cause of exogenous fungal endophthalmitis: postsurgical [3, 4] or posttraumatic [5]. In a study on vitrectomy for endogenous fungal endophthalmitis by Shen and Xu [6],* Aspergillus niger* represented 10% of the cases, while* Candida albicans* was by far the most common organism.

Contrary to other case reports where vitreous biopsy was positive, our case's vitreous biopsy was negative bilaterally. Surprisingly, Subretinal aspirate biopsy yielded positive results and solved the dilemma. Treatment with intravitreal amphotericin B remains the gold standard for fungal endophthalmitis cases. Retinal toxicity should be kept in consideration.

Although systemic workup for the patient did not reveal any abnormality (blood and chest imaging), we think that bilateral choroidal seedling with fungal infection is the result of transient fungemia (which could be related to history of drug abuse). The first vitreous biopsy showed abnormal lymphocytes which, together with B scan evidence of choroidal thickening, pointed to intraocular lymphoma as a possible differential diagnosis, although the clinical presentation and fundus appearance were not typical of lymphoma (subretinal infiltrate was confluent and not scattered as in lymphoma).

While two vitreous biopsies (one from each eye) were negative for microbes, subretinal biopsy was positive. This could be explained by the fact that the organism is seeded from the blood to the choroid and may proliferate underneath the retina with minimal penetration into the vitreous cavity. This was similar to other reports about* Aspergillus* endophthalmitis by Kiang L et al. [7], in which positive culture from retinal invasive aspergilloma confirmed the diagnosis after initial diagnostic vitrectomy yielded a single colony of* Aspergillus* and was initially considered a contaminant. The difference, however, lies in the fact that our vitreous biopsies were negative.

Intraocular lymphoma can masquerade posterior uveitis. The incidence of primary vitreoretinal lymphoma (PVRL) has increased during the last few decades. Differential diagnostic distinction between lymphoma and posterior uveitis is often difficult, so that adequate diagnosis and treatment are often delayed. This is fatal, because PVRL is often associated with primary central nervous lymphoma. To confirm the diagnosis, prior treatment of cytological or histological detection of lymphoma cells is the gold standard. Therefore, a diagnostic vitrectomy should be performed with vitreous biopsy and sometimes transretinal biopsy [8].

## 4. Conclusion

- Subretinal aspirate and retinal biopsy are important armamentarium in the diagnosis of infectious uveitis when vitreous biopsy is negative.
